# Risk Aversion Among Male Older Adults: Does Veteran Status Matter?

**DOI:** 10.1093/geroni/igaa057.2180

**Published:** 2020-12-16

**Authors:** Kent Jason Cheng, Scott Landes, Janet Wilmoth

**Affiliations:** 1 Syracuse University, Syracuse, New York, United States; 2 Aging Studies Institute – Syracuse University, Syracuse, New York, United States

## Abstract

Risk aversion determines how people make decisions and is known to predict a wide array of economic outcomes. This study assessed whether there are veteran status differences in risk aversion utilizing the Health and Retirement Study. Risk aversion is based on hypothetical financial gambles (N=2,121; 2006 wave) and self-reported risk attitudes on selected topics (N= 4,980; pooled 2014 and 2016 waves of the Leave-Behind Survey). Results from multivariate analyses reveal that veterans were more likely to be risk averse than nonveterans in financial matters, occupation, and health, but veteran status is not statistically significant in explaining risk taking in driving and leisure, and sport risk. Further research is needed to discern the role of military service-related experiences in determining levels of risk aversion among veterans and the extent to which risk aversion accounts for veteran status differences in later-life economic outcomes. Part of a symposium sponsored by the Aging Veterans: Effects of Military Service across the Life Course Interest Group.

